# Involvements of Hyperhomocysteinemia in Neurological Disorders

**DOI:** 10.3390/metabo11010037

**Published:** 2021-01-06

**Authors:** Marika Cordaro, Rosalba Siracusa, Roberta Fusco, Salvatore Cuzzocrea, Rosanna Di Paola, Daniela Impellizzeri

**Affiliations:** 1 Department of Biomedical, Dental and Morphological and Functional Imaging, University of Messina, Via Consolare Valeria, 98125 Messina, Italy; cordarom@unime.it; 2 Department of Chemical, Biological, Pharmaceutical and Environmental Sciences, University of Messina, 98166 Messina, Italy; rsiracusa@unime.it (R.S.); rfusco@unime.it (R.F.); dimpellizzeri@unime.it (D.I.); 3 Department of Pharmacological and Physiological Science, Saint Louis University School of Medicine, Saint Louis, MO 63104, USA

**Keywords:** homocysteine, hyperhomocysteinemia, target, physiology, pathology

## Abstract

Homocysteine (HCY), a physiological amino acid formed when proteins break down, leads to a pathological condition called hyperhomocysteinemia (HHCY), when it is over a definite limit. It is well known that an increase in HCY levels in blood, can contribute to arterial damage and several cardiovascular disease, but the knowledge about the relationship between HCY and brain disorders is very poor. Recent studies demonstrated that an alteration in HCY metabolism or a deficiency in folate or vitamin B12 can cause altered methylation and/or redox potentials, that leads to a modification on calcium influx in cells, or into an accumulation in amyloid and/or tau protein involving a cascade of events that culminate in apoptosis, and, in the worst conditions, neuronal death. The present review will thus summarize how much is known about the possible role of HHCY in neurodegenerative disease.

## 1. Introduction

Homocysteine (HCY) is a sulfhydryl-containing amino acid produced following the demethylation of methionine, an amino acid contained primarily in animal protein. To recondition methionine, HCY is recuperated using a pathway that requires folic acid and vitamins B6/B12 [1]. Pathologically high blood levels of HCY, indicated as hyperhomocysteinemia (HHCY), signal a breakdown in this biochemical process, resulting in biochemical and life consequences [2]. Variations in HCY metabolism can also be developed with certain dietary and lifestyle modifications such as increased coffee drinking, cigarette smoking, and alcohol abuse, as these interfere with methionine synthase activity [2]. Until today, HHCY has been epidemiologically and clinically correlated in a variety of pathological conditions and was confirmed also as an independent risk factor for many different pathologies such as cardiovascular disease, neural tube defects, osteoporosis, neuropsychiatric disorders and many, many, others [3,4,5,6,7,8,9,10,11,12,13,14,15,16,17,18,19,20,21,22,23]. Recent evidence, additionally, related high levels of HCY as a factor that contributes to the development of certain cancers [24,25,26]. Considering the literature, most of the information of HHCY-related pathologies were focused on cardiovascular disease but less was analyzed for brain disorder. With these aims in mind, we studied the recent literature of HHCY and brain disorders.

## 2. Physiological Role of Homocysteine

### 2.1. Cycle

**Figure 1:**
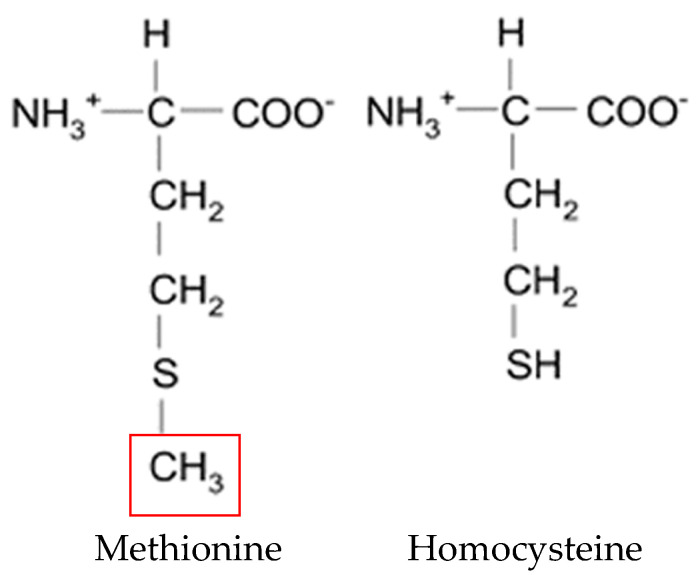
Chemical structure of methionine and homocysteine.

HCY is a physiological metabolite of the essential amino acid methionine (Figure 1). Considering that nutriment contains little or no HCY, nearly all of the HCY in the body is derived from the methionine contained in animal and plant proteins [27,28].

**Figure 2:**
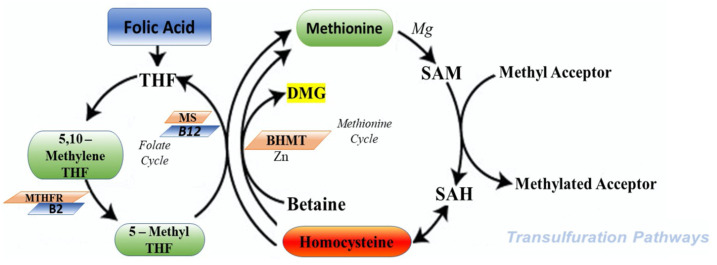
Transsulfuration cycle. The metabolism of HCY (homocysteine) is composed by two different steps. MS—methionine synthase; MTHFR—methylene tetrahydrofolate reductase; BHMT—betaine HCY methyltransferase; DMG—dimethylglycine; B2—riboflavin; THF—thetrahydrofolate; SAM S—adenosyl methionine; SAH S—adenosyl homocysteine.

**Figure 3:**
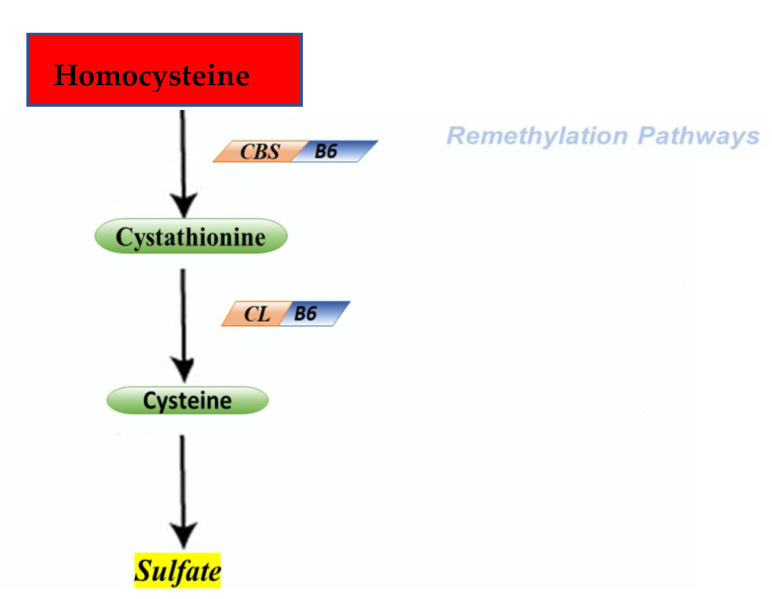
Remethylation pathways. Second step is represented by remethylation of homocysteine. CBS—cystathionine – β – synthase; CL—cystathionine – γ – lygase.

The cycle of HCY is composed by two different pathways that intersect with each other in two points during transsulfuration (Figure 2) and remethylation (Figure 3).

During the transsulfuration pathway, HCY condenses with serine to form cystathionine in a non-reversible reaction catalyzed by the pyridoxal-5′-phosphate (PLP)-containing enzyme, cystathionine β-synthase. In the next step, cystathionine is hydrolyzed by a second PLP-containing enzyme, γ-cystathionase, to form cysteine and α-ketobutyrate. Cysteine, in surplus, is oxidized to taurine or inorganic sulfates or is excreted in the urine. Consequently, in addition to the physiological synthesis of cysteine, this transsulfuration pathway effectively catabolizes excess HCY, which is not required for methyl transfer.

On the other hand, during remethylation, HCY, to form methionine, acquires a methyl group from N-5-methyltetrahydrofolate or from betaine. This metabolic reaction with N-5-methyltetrahydrofolate happens in all tissues and is vitamin B12 dependent; however, the reaction with betaine is restricted mainly to the liver and is not dependent by vitamin B12. The most part of methionine is successively activated by ATP to form S-adenosylmethionine (SAM). SAM have a key role to donate a methyl group to different acceptors. At the end of this pathway, the S-adenosylhomocysteina (SAH) produced is, in the last step, hydrolyzed, thus restoring homocysteine, which then becomes accessible to start a new cycle.

Considering that this hydrolysis is a reversible reaction that favors the synthesis of SAH, it is important to say that probably the elevated cellular concentrations of this metabolite are the first step to precede and accompany all forms of hyperhomocysteinemia [29].

### 2.2. Circulation and Concentration of HCY in Human Body

Almost 3% of physiological levels of HCY circulate freely in the body in bound form with other molecules or in disulfide form [30,31]. The majority of HCY in the plasma is in disulfide form and is oxidized by reacting with other molecules that contain free thiol groups such as albumin, and the remaining exists as a reduced form. Pathological factors such as a deficiency in folate and cobalamin levels, or mutations and polymorphisms in key enzymes in the metabolic pathway, such as MS, *MTHFR*, and cystathionine β –synthase (CBS) are straightly correlated with elevated levels of HCY [29,32,33,34]. In addition, the decreased folate carrier involved in the influx of 5-MTHF cells is associated with low folate, changes in the pattern of DNA methylation, and DNA repair ability [35,36].

In addition to genetic changes, vitamin shortages, and many other environmental factors such as elevated Met intake, it is understood that some drugs, cancer status, breastfeeding, and lactation lead to fluctuations in HCY levels [37,38,39,40,41,42].

It is also established that altered cellular export pathways raise the levels of HCY [43]. In general, considering its low level in most foods, the dietary contribution of HCY alone is insignificant, mainly derived from Met [44].

In human plasma, in physiological conditions, HCY concentration is below 12–15 μM and the cysteine concentration level comprises between 240 and 360 μM [45]. There was no difference between male and female high levels of HCY; on the other hand, there was a gender difference in HCY metabolism and low concentration of vitamin B12 and folate in males.

According to HCY levels in plasma, HHCY is considered: severe (>100 μmol/L), intermediate (31–100 μmol/L) or moderate (16–30 μmol/L) [46]. It is mandatory to export the excess of HCY from the intracellular environment into the systemic circulation. The liver and kidneys are responsible for its clearance because of BHMT and CBS, which convert HCY into nontoxic metabolites [47].

## 3. Pathological Role of Homocysteine

Most accreditable hypotheses of HCY-induced cell damage is related to oxidative stress [48,49,50]. Pre-clinical and clinical studies demonstrated that HCY perturb mitochondrial function at several different levels, in particular, disturbing oxidant/antioxidant systems [51,52,53]. In energy generation, mitochondria play an important role through the electron transport chain (ETC) coupled with oxidative phosphorylation (OXPHOS), the tricarboxylic acid cycle (TCA) and the β-oxidation of fatty acids [54]. It is accurately, at this point, that HHCY decreased mitochondrial respiration associated with reduced activities of ETC complexes and diminished ATP production [55]. In particular, different studies demonstrated that HCY inhibits complex I activity in the cerebral cortex of immature rats or ETC complexes I, IV, and V in hippocampus, complex IV in amygdala, and complexes IV and V in cerebral cortex [56,57,58,59,60,61,62]. Obliviously, perturbing mitochondrial function was followed by a modification in physiological antioxidant system. The increased activity of cytosolic Cu/ZnSOD and catalase in the nucleus caudatus putamen and substantia nigra was associated with chronic HHCY in mice. Similarly, chronic mild HHCY in rats increased the amygdala and prefrontal cortex activity of SOD, catalase, and GPx. Counteracting ROS production through the upregulation Nrf2 were correlated with increases in antioxidant defense in these tissues. On the other hand, in the cortex and hippocampus of rats persistently treated with HCY, MnSOD activity was shown to be unchanged [56,58,60,63].

The key role of HCY is a biochemical juncture between the metabolism of methionine and the biosynthesis of cysteine [64,65]. For these reasons, HCY metabolism is closely regulated by the different similarities between methionine synthase and cystathionine β–synthase for homocysteine, so methionine conservation is preferred at low HCY concentrations. On the other hand, instant and long-term drainage of HCY through the trans-sulfuration pathway is ensured when HCY concentrations are increased [66].

An excessive elevation of HCY in plasma and urine can be caused by many congenital and nutritional disorders, as well as renal failure, and this represents an imbalance between HCY production and metabolism [67,68].

Deficiencies in vitamin B12, folate and vitamin B6 are nutritional deficiencies that potentially contribute to deficiency of HCY metabolism, as the de novo synthesis of methionine methyl groups involves both vitamin B12 and folate co-factors, whereas the synthesis of cystathionine requires pyridoxal 5′-phosphate (vitamin B6) [69,70,71,72,73,74,75].

Meta-analysis studies demonstrated that a daily folic acid supplementation of about 0.5–5 mg contributes to a reduction of HHCY by around 25%. Vitamin B12 (0.5% extra) decreases the concentration of HCY by another 7% [64,65]. Another research found that vitamin B12 and folate supplementation decreased the levels of HCY by 7% and 23% respectively [76]. Vitamin B6, vitamin B12, and folate, respectively, were seen to decrease by 12%, 5%, and 43% in HCY level [77,78].

HCY was considered as a problem especially during HHCY condition which is associated with many medical problems. Up until October 2020, more than 9200 works on PubMed link HHCY with several different pathologies (Table 1).

### 3.1. Alzheimer’s Disease

Clinical data reports indicate that HHCY is an independent risk factor for the transformation of a healthy cognitive individual to dementia in both normal elderly and Alzheimer’s disease individuals [79]. HHCY is a condition developed with or without mild cognitive impairment (MCI) in healthy individuals. A relationship between learning and hippocampal activity and HHCY indicates that by inducing brain atrophy in patients with MCI, HHCY degrades cognitive functions in both healthy controls and MCI patients [80,81,82].

Another study shows a connection between homocysteine, hippocampal plasticity and synaptic transmission, indicating shortcomings in learning and memory [80,81,82]. The auto-oxidation of HCY that leads to the formation of ROS that lead to neuroinflammation and apoptosis could explain the neurotoxicity caused by HCY [83]. HHCY has been reported to modify the structure and function of cerebral blood vessels through oxidative stress and endothelial dysfunctions, leading to impairment of perfusion accompanied by neuronal disorders and marked as risk factors in vascular dementia and Alzheimer’s disease pathogenesis [84].

The identification of the mechanism that linked HHCY and dementia has garnered a lot of interest. It is suspected to function as an excitatory neurotransmitter that competes with the gamma-aminobutyric acid (GABA) inhibitory neurotransmitter. In addition, through inducing microvascular permeability, it inhibits GABA-A/B receptors, which then increases redox tension, which further activates disintegrin and metalloproteinase, thereby terminating metalloproteinase tissue inhibitors. The blood–brain barrier matrix contributing to vascular dementia is broken [85].

Zhang et al. reached the conclusion that increases in plasma HCY levels may induce amyloid-beta peptide acquisition and increase Alzheimer’s like tau phosphorylation in rats as well. HCY has also been found to make neurons prone to amyloid-beta toxicity and it tarnishes the DNA repair process in hippocampal neurons [86].

### 3.2. Parkinson’s Disease

Clinical studies have shown that HHCY has been observed in Parkinson’s disease patients, who may also be involved in Parkinson’s disease pathogenesis [81]. Microglia and astrocytes that cause an inflammatory response that causes neuronal death can be activated by HCY [87]. The substantia nigra region is found to be inflammatory in patients with Parkinson’s disease, and inhibition of this inflammation has proven to be neuroprotective in the Parkinson’s disease model. The inflammation is probably due to the NO released that leads to an activation of microglia and astrocytes, which shows harmful effects on neurons resulting in neurodegeneration. NO release can be determined in rodents by following the MPTP or 6-hydroxydopamine (6-OHDA) model and by comparing neuronal death when only 6-OHDA is given and when co-administered with a NO scavenger [88].

Mitochondrion is a site where different neurodegenerative diseases, including Parkinson’s disease, are investigated for pathogenesis. By means of electron microscopy, HCY was found to cause swelling of mitochondria inhibited by the binding of Cyclosporin A to the mitochondrial matrix protein Cyclophilin D, thereby blocking the formation of mitochondrial permeability transition (MPT) calcium-dependent [89]. This study links HCY directly to mitochondrial disruption, which ultimately leads to neuronal loss in Parkinson’s disease. Levodopa, the most prevalent treatment for Parkinson’s disease, is complicated by HCY levels. It induces HHCY via catechol-O-methyltransferase (COMT) due to its methylation. This complication, since it is a COMT enzyme inhibitor, can be cured by treating patients with entacapone. However, even after all these studies considering HHCY as an independent risk factor for Parkinson’s disease, further study still needs to be done to prove the assumption [90].

There is still confusion because the HCY-neuronal cell death pathways merge at a point, i.e., oxidative stress, which can both cause HHCY and be an outcome of HHCY, making it difficult to decide which first occurs. In this confusing relationship between animal models of HHCY and Parkinson’s disease, antioxidants have helped to reduce the effects of HCY and are also found to reduce the effect of bone loss due to Parkinson’s disease [91,92].

### 3.3. Autism

Autism spectrum disorders (ASD) are an heterogeneous class of repetitive habits, limited desires, gastrointestinal and immunological comorbidities associated with neurodevelopmental disorders that occur before 3 years of age that often lead to social and language compromises. In the United States, the real occurrence is 1 in 68 girls, with a 4:1 male to female prevalence [93].

While autistic behavior can occur in many chromosomal, genomic, monogenic, dysmorphic, and metabolic syndromes (microdeletions, insertions, and imprinting), most cases are multifactorial in origin, with some susceptibility loci [94,95,96]. In different cases, brain dysfunction can be linked, to some degree, with hypomethylation of the subcellular portion and oxidative stress injury, both pathogenic mechanisms involving altered HCY metabolism as an associated cause, as mentioned above [94,95,96].

In 2004, James and colleagues reported a distorted remethylation of HCY to methionine and transsulfuration of HCY to cysteine in children with autism [97]. The metabolic phenotype of children with autism highlighted decreased plasma concentrations of methionine, SAM, HCY, cysteine, and total GSH, and increased concentrations of SAH, adenosine, and oxidized GSH compared to control infants [97].

Opposite findings were published by Tu et al. in China and Ali et al. in Oman, where, relative to an age- and gender-matched control sample, children with autism presented elevated plasma HCY levels. Reduced plasma folate concentrations in children with autism have been seen in these trials, and in the study by James et al., the cases tested were treated with folic acid and vitamin B12, which may explain the various outcomes [97,98]. Additionally, in comparison to controls, Ali et al. have found decreased plasma vitamin B12 concentration in some cases [99].

Increased urinary concentration of HCY was also found in Poland for non-supplemented children with autism [100]. Studying HCY metabolism in multiple subtypes of ASD showed a deficiency of this metabolic mechanism in more serious cases across non-specified pervasive conditions and prototypic autistic disorder with enhanced metabolic derangement. In moderate cases, there was only remethylation dysfunction but transsulfur disruptions were present in the most extreme cases. Additionally, in altered HCY metabolism in autism, dietary factors may also be involved, in consideration of food intake problems with differing intake and consequent unbalance of protein and vitamins, but genetic polymorphisms can also be important in genes involved in this metabolic pathway, and therefore, it is mandatory to be evaluated [101,102,103].

### 3.4. Schizophrenia

Schizophrenia is a severe multifactorial psychiatric disorder, sometimes disabling, that affects 1% of the world’s population [104] with the occurrence of positive symptoms such as hallucinations, hysteria and delusions or negative symptoms such as reduced energy, impoverished expression, blunted effect and social withdrawal [105,106]. In 1995, Regland et al. were the first to link an increase in HCY blood levels with schizophrenia [107,108]. In 2006, the correlation was subjected to a meta-analysis by Muntjewerff et al., who gathered evidence from eight case-control trials and found a 70% rise in the probability of schizophrenia with every 5 mM increase in HCY concentration, and several other studies have since validated this hypothesis, while negative findings are also present in the literature [109]. HCY metabolism-related genetic factors are also associated with the risk of schizophrenia. In this association, dietary variables may also play a part. Increased HCY levels and schizophrenia are often linked with low folate levels [110,111,112]. Low betaine plasma levels have also been seen in first-episode schizophrenia patients and can affect the metabolism of HCY in these people [110,111,112]. In addition, the connection between HCY metabolism and schizophrenia can also be connected with fetal hypoxia, impaired DNA methylation, and selective antagonistic effects on N-Methyl-D-aspartate (NMDA) glutamatergic neurons [110,111,112,113,114,115].

In patients with schizophrenia, few experiments have been conducted on HCY-lowering interventions. A multicenter randomized clinical trial was reported by Roffman et al., in which a 16-week supplementation of folate and vitamin B12 resulted in improved negative symptoms in chronic patients, measured by the Scale for Evaluation of Negative Symptoms and the Positive and Negative Syndrome Scale [105,116,117].

### 3.5. Major Depressive and Bipolar Disorder

Major depressive disorder (MDD) is a serious and complex psychiatric disease characterized by anhedonia, depression and significant distress. The cause of MDD is associated with changes in brain neuroanatomy, neurotransmitters and neuroendocrine systems, along with strong evidence for genetic factors [118,119,120]. MDD is currently the third largest debilitating disease, according to the World Health Organization (WHO), affecting 1% to 2% of pre-adolescent children and 0.9% to 42% of geriatric persons in the Caucasian population [121].

Actual evidence for the link between depression and HCY comes from multiple trials of patients with depression that find elevated levels of HCY [121,122]. In addition, folate deficiency has been observed in up to one-third of extreme depression patients [123]. It is important to remember that evaluations discussing this issue have contradictory findings, as most trials evaluating HCY levels are conducted in elderly patients and both HCY levels and the onset of depression with aging have increased [120,121]. Folate deficiency is also due to inadequate nutrition in these patients. Moreover, some medications used for stress relief can potentially interact with the synthesis of folate and HCY [124]. However, whether the deficiency is primary or secondary to depression, low folate levels limit the exposure to antidepressants [125].

Bipolar disorder, also referred to as an idiopathic personality disorder, is characterized by depression and mania episodes and affects between 2% and 4% of the global population [165,166,167,168]. High levels of HCY can potentially be harmful to dopaminergic processes, and bipolar disorder has been linked with dysfunction of dopamine neurons [126,127]. In addition, increased HCY concentrations and decreased folate and vitamin B12 concentrations are found in both acute episode and euthymic period patients with bipolar depression; low appetite observed in these patients may be linked with reduced vitamin B intake and consequent HHCY [128,129]. Nevertheless, the mechanisms underlying HHCY in bipolar disorder are not well known and tend to include not only food intake, but also diminished glomerular filtration and mood-stabilizing drug utilization [124,130,131,132,133]. Some treatment used in these pathologies, such as valproic acid and lamotrigine, can interfere with folate and HCY metabolism through methionine adenosyltransferase and dihydrofolate reductase inhibition [134]. Baek et al. proposed that folate supplementation could normalize monoamine synthesis and adjust mood stabilizer-associated functional folate deficiency because improved levels of HCY are observed in bipolar patients, and folate is a co-factor involved in both HCY metabolism and monoamine synthesis [124,130].

### 3.6. Vascular Dementia

Vascular dementia (VD) is the most prevalent type of dementia after Alzheimer’s disease, which, based on demographic, age, and diagnosis criteria, accounts for 40 percent of dementia cases. Vascular dementia is also known as multi-infarct dementia, since dementia is caused by many minor brain infarctions [110,111,112,135,136,137,138,139]. In a study of 27 HHCY patients compared to 98 normal controls, Evers et al. found a significant increase in blood pressure and microangiopathy in the hyperhomocysteinemic group, as well as a trend towards a higher rate of multiple infarctions [140].

This link between HCY levels and dementia was not observed in the Rotterdam Study, but it suggested that patients with cognitive disability were older, less educated, and had a higher incidence of vascular disease and stroke [114,140]. This prompted the authors to state that HCY may cause vascular damage leading to cognitive decline; however, their analysis had a follow-up duration of just 2.7 years, which could have been too short a period of time to detect a difference in the MMSE because of elevated HCY levels. [114,140].

### 3.7. Ischemic Stroke

A stroke is the primary factor for adult injury and the second leading cause of death worldwide. Stroke is either ischemic or hemorrhagic, which, by rupturing blood vessels, disrupts the blood flow to part of the brain. Clinical trials have shown that HHCY is a predictor of stroke and thrombophilia linked to stroke. Via the mechanism of attenuated anticoagulant processes, increased thrombin production, impaired enzyme breakdown, and attenuated anticoagulant processes, there is a rapidly accumulating association between HHCY and thrombosis [145].

8-iso-prostaglandin f2-α, a marker for oxidative stress signaling high lipid peroxidation due to platelet activation, has been observed in HHCY patients with homozygous CBS deficiency [145,146,147,148].

HHCY also involves ocular damage; redundancy of non-arteritis anterior ischemic optic neuropathy and CBS deficiency have been identified, causing retinal embolism due to craniocervical arterial dissection [80,81]. In context, oxidative stress and reduced fibrinolytic capacity in experimentally-induced HHCY revealed that in rat cortex and hippocampus, an elevated level of HCY substantially improves cell neurodegeneration.

In ischemic stroke patients with no internal carotid arterial stent-occlusion (ICS), a relationship in the amount of rise of plasma HCY levels and pulsatility index in all intracranial arteries came into observation [149]. These studies bring to a conclusion that HHCY is a mediator for aortic plaque development. HHCY affects intracellular signaling in ischemia-induced neurodegeneration along with ischemic preconditioning [149].

Ischemic preconditioning represents adaptation of the CNS to sub-lethal ischemia, resulting in increased brain tolerance to subsequent ischemia. Until today, the knowledge about effects of HCY and ischemic preconditioning (IPC) in animal models of ischemic stroke is very poor [149]. Blaise et al. showed that by inducing neurogenesis, short hypoxia could inhibit the deleterious effects of HHCY on rat brain growth [169]. As a type of preconditioning, brief neonatal hypoxia significantly promoted the migration of new neurons to permissive areas such as the subventricular and hippocampal areas, improved locomotor control and memory and learning, and attenuated the long-term effects of HHCY. Similarly, as a type of preconditioned pulse, physical activity has a beneficial effect on HHCY -induced seizures [170]. According to the scientists, this reduces susceptibility to seizures, which is at least partially the product of increased activity of antioxidant enzymes [149].

### 3.8. Epilepsy

Epilepsy is a neurological condition that arises due to the irregular firing of prefrontal cerebral nerves, resulting in repetitive and unconscionable seizures. Convulsions, hypertonic and stereotyped gestures, changes in beliefs and feelings, and unconsciousness can be the measurable symptoms of seizures. HHCY is seen in epilepsy cases, but in addition to epilepsy, there may also be several other dominant causes, such as adverse effects caused by long-term use of anti-epileptic medications (carbamazepine, gabapentin, phenytoin, primidone, valproate, and oxcarbazepine), particularly in epileptic patients, that may be responsible for the production of hyperhomocysteinemia [150]. In MRI studies of 58 epilepsy patients with HHCY, Gorgone et al. observed a greater rate of brain atrophy along with being on antiepileptic medications. From these findings, he suggested that in patients with epilepsy, both HHCY and polypharmacy confer brain atrophy [151,152]. Higher amounts of homocysteic acid and HCY sulfinic acid are found to display excitotoxicity by both NMDA (N-methyl-D-aspartate) and non-NMDA receptors in juvenile epilepsy cases of homocystinuria. HHCY also ceases glutamate decarboxylase operation and interrupts glutamine metabolism [153].

The possibility that high doses of HCY causes seizures when delivered systemically in animals is heavily used in laboratory epilepsy models. Another fact found is that about 20 percent of patients with homozygous CBS deficiency undergo seizures that may develop into epilepsy when combined with elevated plasma HCY concentrations of generally 50–200 μmol/L. However, it is not yet confirmed that lower plasma HCY levels ranging from 15 and 20 μmol/L lead to epilepsy in patients [154].

### 3.9. Peripheral Neuritis

Peripheral neuritis is an elderly condition that, based on the nerve fibers affected, has different signs of visual, motor and autonomic functional imbalances. The different etiologies found to date include metabolic diseases, infections, inflammation, malnutrition mediated by autoimmune, inherited conditions, and unique drug and radiation toxicities. Clinical trials have shown that HHCY in diabetic patients raises the incidence of peripheral neuropathy and worsens the pre-existing condition of diabetic neuropathy in peripheral neuritis patients [141].

Earlier pig model experiments demonstrated an elevation of the adenosyl HCY (AdoHCY) level in methyl deficit neuronal tissues and indicated that this elevated AdoHCY level induces potentially treatable peripheral neuropathy [80,81,142,143,144].

### 3.10. Headache

HHCY may lead to cerebral flow modification, with the possibility of thrombosis and cerebral oxygen transfer alterations, eventually facilitating migraine aura events [155]. Kara et al. have shown that the C677T polymorphism on the *MTHFR* gene can affect migraine susceptibility, with migraine due to the increase in HCY levels in the blood as reported above [156]. Cacciapuoti demonstrated that in individuals usually suffering from migraine—especially migraine with aura—an increased serum level of HCY may be present [157]. Among the reasons could be vasodilation or transient thrombosis in HCY-induced cerebral blood vessels [156,171]. In addition, enhanced serum levels of HCY are responsible for a hypercoagulable condition supported by elevated von Willebrand factor or prothrombin activation [158,159,160,161]. The correlation between HCY and hypercoagulation may also explain the increased risk in these patients of stroke and cardiovascular events [172]. In addition, through the formation of superoxide anions, HCY may play a role in the onset of migraine for oxidative damage to the vascular endothelium [162,163,164].

Additionally, the only study assessing homocysteine in cerebrospinal fluid reported that the concentration of this biomarker was substantially elevated in migraine patients relative to controls. Although this data certainly needs to be validated in larger research, certain putative mechanisms may encourage a causal link between increased brain homocysteine generation and migraine [173].

### 3.11. Multiple Sclerosis

The relationship between elevated blood levels of homocysteine levels and the incidence of vitamin folate or B12 deficiency in patients with multiple sclerosis (MS) has motivated the analysis of homocysteine levels in MS. Vitamin B12 or folate deficiencies, cofactors in the intracellular transformation of homocysteine into methionine, contribute to elevated plasma or serum homocysteine [174,175,176,177].

Homocysteine could also directly affect CNS cells or influence the activation of macrophages, essential aspects of MS pathology [176,178,179]. Recent studies have shown that MTHFR gene coding mutations, the key recognized genetic determinant of elevated homocysteine levels, were not overrepresented in MS patients [180,181,182]. Interestingly, the results of the few studies carried out so far on serum or plasma levels in MS indicate that homocysteine levels may increase compared to healthy controls in patients with MS, whereas no changes in vitamin B6/B12 or folate status have been reported. No variations between clinical subtypes of MS were observed in total homocysteine levels. Interestingly, a recent study noted a link between cognitive functioning and homocysteine, which affects as many as 30–70% of MS patients [183,184,185,186,187,188,189,190,191,192,193,194,195,196,197].

Teunissen and colleagues, in particular, have studied the relationship between serum homocysteine levels in patients with different MS subtypes and related homocysteine levels to cross-sectional and longitudinal parameters of clinical disease progressions, and found that serum homocysteine levels are straightly related to disease progression [195]. This is probably due to the role of the transmethylation pathway in MS [195]. The most accredited hypothesis is based on hypomethylation of myelin basic protein (MBP)-arginine that decreases the hydrophobicity of MBP and could give rise to less stable myelin structures and enhance degeneration of the myelin sheath [175,188,195]

### 3.12. Neurodegeneration and Neurovascular Disorders in Diabetes

Neurovascular and neurodegenerative disorders are common in patients with diabetes mellitus (DM) and risk factors such as HHCY, seemingly unrelated to diabetes, can be attributed to the atherothrombotic mechanism in these subjects. Plasma homocysteine levels are typically common for diabetes, but both lower and higher levels have been recorded. This has been due to hyperfiltration and renal impairment or reduced folate status, respectively. Resistance to insulin does not seem to be a significant determinant of plasma homocysteine levels. Microalbuminuria and retinopathy have been linked with HHCY for Type 1 and Type 2 diabetes. Concentration of plasma homocysteine in patients with Type 2 diabetes has also been found to be associated with macrovascular disease and death. This association tends to be greater in diabetics than in diabetes-free subjects. The underlying pathophysiological cause of this elevated vascular risk remains unexplained but may contribute to deteriorating endothelial dysfunction or properties of systemic vessels. The DM pathobiological pathway leading to peripheral and/or autonomic neuropathy is complex and not fully understood. Homocysteine leads to neuropathy by functioning as a direct neurotoxic factor or causing neurovascular dysfunction. An association between the amount of plasma homocysteine and the existence of autonomic or peripheral neuropathy has actually been identified in several studies investigating this link [198,199,200], however, other studies could not establish such a relationship [201,202,203].

Only two experimental studies were set up to identify predictors of diabetic neuropathy (in Type 2 diabetes). Additionally, to correct other variables, multivariate analysis was conducted. It produced conflicting results, with one study discovering an association between neuropathy and homocysteine [200] and not the other [203].

Therefore, the proposed role of HHCY in the development of diabetic neuropathy cannot be ignored, but further research is required. Different evidences indicate that HHCY plays a significant role in diabetic patients in inducing retinal ganglion cell apoptosis. HHCY and vitamin B12 deficiency, in particular, have been shown to have a role in diabetic retinopathy.

In both in vitro and in vivo models, increased homocysteine levels have been shown to cause retinal ganglionic cell apoptosis. In vitro studies of RGC cells and in vivo brain studies indicate that homocysteine acts as an NMDA receptor glutamate site agonist [204,205].

Other studies have also shown that homocysteine’s neurotoxic effects are also correlated with the activation of glutamate type II receptors. Potential treatment options to boost neurodegeneration can also be strategies to regulate the amount of homocysteine through supplementation with folic acid or vitamin B12.

The neuroprotective agent developed in the kynurenine pathway is kynurenic acid (metabolic degradation pathway of tryptophan). Excitotoxicity induced by the glutamate receptor and free radical development are also shown to be related to the neuroprotective metabolite kynurenic acid. The development of kynurenine [206] may also be influenced by homocysteine. It has been proposed that the adverse effects of high homocysteine levels on the supply of kynurenic acid [207] are further enhanced by hyperglycemia.

## 4. Conclusions

This review aims to summarize several findings on possible links between HHCY and different brain pathologies.

Metabolism/catabolism of methionine and HCY are based on complex biochemical pathways involving the co-operation of multiple enzymes and producing various molecules that are essential biochemical steps for cell survival.

Interestingly, HHCY is currently not only seen as the diagnostic marker for pathologies, but a possible therapeutic target is also considered. A diet deficient in folic acid, vitamin B6, vitamin B12, and betaine has been reported to be responsible for developing HHCY. Consequently, being able to compensate for the shortcomings of these important components in clinical practice must be considered to be of high therapeutic relevance. The administration of folate, group B vitamins and other molecules entering the metabolic cycle of methionine has been reported in several studies to reduce the severity of HHCY, helping in several pathological conditions and also in pregnancy.

The HCY level imbalance has a lot to do with different cognitive diseases. HCY synthesis disturbance is a source of redox impairment due to the formation of reactive oxygen and nitrogen species, which are, again, the basis for the pathogenesis of different neurological diseases. HCY is known to amplify amyloid beta deposition, modify presenilin functions and is also found to restrain tau protein hyperphosphorylation in Alzheimer’s and dementia. Elevated levels of HCY are an early marker for the disease, in addition to carotid atherosclerosis or white matter lesion. The relationship between HHCY and cognitive impairment is clinically proven through amyloid deposition and hyperintensity of white matter. This review is intended to be a summary of several pieces of evidence that can show how HHCY is involved in several pathologies, and while it is far from being considered a biomarker of these pathologies, clinical interventions can still be a good target.

## Figures and Tables

**Table 1:** Pathology related to high HCY levels. This table contains a recent bibliography of the most common brain disorders related to the high HCY levels.

Pathology	Reference
Alzheimer’s Disease	[79,80,81,82,83,84,85,86]
Parkinson’s Disease	[81,87,88,89,90,91,92]
Autism	[93,94,95,96,97,98,99,100,101,102,103]
Schizophrenia	[104,105,106,107,108,109,110,111,112,113,114,115,116,117]
Major Depressive and Bipolar Disorder	[118,119,120,121,122,123,124,125,126,127,128,129,130,131,132,133,134]
Vascular Dementia	[110,111,112,114,135,136,137,138,139,140]
Peripheral Neuritis	[80,81,141,142,143,144]
Stroke	[80,81,145,146,147,148,149]
Epilepsy	[150,151,152,153,154]
Headache	[155,156,157,158,159,160,161,162,163,164]

## References

[1] Kok F.J. (2001). Folic acid, vitamins B6 and B12: Relation to homocysteine and cardiovascular disease. Bibl. Nutr. Dieta.

[2] Miller A.L. (2003). The methionine-homocysteine cycle and its effects on cognitive diseases. Altern. Med. Rev..

[3] Hopkins P.N., Wu L.L., Wu J., Hunt S.C., James B.C., Vincent G.M., Williams R.R. (1995). Higher plasma homocyst(e)ine and increased susceptibility to adverse effects of low folate in early familial coronary artery disease. Arterioscler. Thromb. Vasc. Biol..

[4] Loehrer F.M., Angst C.P., Haefeli W.E., Jordan P.P., Ritz R., Fowler B. (1996). Low whole-blood S-adenosylmethionine and correlation between 5-methyltetrahydrofolate and homocysteine in coronary artery disease. Arterioscler. Thromb. Vasc. Biol..

[5] Boushey C.J., Beresford S.A., Omenn G.S., Motulsky A.G. (1995). A quantitative assessment of plasma homocysteine as a risk factor for vascular disease. Probable benefits of increasing folic acid intakes. JAMA.

[6] Robinson K., Mayer E.L., Miller D.P., Green R., van Lente F., Gupta A., Kottke-Marchant K., Savon S.R., Selhub J., Nissen S.E. (1995). Hyperhomocysteinemia and low pyridoxal phosphate. Common and independent reversible risk factors for coronary artery disease. Circulation.

[7] Petronijevic N.D., Radonjic N.V., Ivkovic M.D., Marinkovic D., Piperski V.D., Duricic B.M., Paunovic V.R. (2008). Plasma homocysteine levels in young male patients in the exacerbation and remission phase of schizophrenia. Prog. Neuropsychopharmacol. Biol. Psychiatry.

[8] Ghosh K., Khare A., Shetty S. (2007). Fasting plasma homocysteine levels are increased in young patients with acute myocardial infarction from Western India. Indian Heart J..

[9] Nikfardjam M., Graf S., Hornykewycz S., Zorn G., Huber-Beckmann R., Wojta J., Huber K. (2001). Homocysteine plasma levels in young patients with coronary artery disease. Relation to history of acute myocardial infarction and anatomical extent of disease. Thromb. Res..

[10] Targher G., Zenari L., Bertolini L., Falezza G., Muggeo M., Zoppini G. (2001). Plasma total homocysteine levels are associated with von Willebrand factor, soluble intercellular adhesion molecule-1, and soluble tumor necrosis factor-alpha receptors in young type 1 diabetic patients without clinical evidence of macrovascular complications. Diabetes Care.

[11] Landgren F., Israelsson B., Lindgren A., Hultberg B., Andersson A., Brattstrom L. (1995). Plasma homocysteine in acute myocardial infarction: Homocysteine-lowering effect of folic acid. J. Intern. Med..

[12] Ishihara J., Iso H., Inoue M., Iwasaki M., Okada K., Kita Y., Kokubo Y., Okayama A., Tsugane S., Group J.S. (2008). Intake of folate, vitamin B6 and vitamin B12 and the risk of CHD: The Japan Public Health Center-Based Prospective Study Cohort I. J. Am. Coll. Nutr..

[13] Chasan-Taber L., Selhub J., Rosenberg I.H., Malinow M.R., Terry P., Tishler P.V., Willett W., Hennekens C.H., Stampfer M.J. (1996). A prospective study of folate and vitamin B6 and risk of myocardial infarction in US physicians. J. Am. Coll. Nutr..

[14] Knekt P., Alfthan G., Aromaa A., Heliovaara M., Marniemi J., Rissanen H., Reunanen A. (2001). Homocysteine and major coronary events: A prospective population study amongst women. J. Intern. Med..

[15] Whincup P.H., Refsum H., Perry I.J., Morris R., Walker M., Lennon L., Thomson A., Ueland P.M., Ebrahim S.B. (1999). Serum total homocysteine and coronary heart disease: Prospective study in middle aged men. Heart.

[16] Bots M.L., Launer L.J., Lindemans J., Hoes A.W., Hofman A., Witteman J.C., Koudstaal P.J., Grobbee D.E. (1999). Homocysteine and short-term risk of myocardial infarction and stroke in the elderly: The Rotterdam Study. Arch. Intern. Med..

[17] Brattstrom L., Lindgren A., Israelsson B., Malinow M.R., Norrving B., Upson B., Hamfelt A. (1992). Hyperhomocysteinaemia in stroke: Prevalence, cause, and relationships to type of stroke and stroke risk factors. Eur. J. Clin. Investig..

[18] Perry I.J., Refsum H., Morris R.W., Ebrahim S.B., Ueland P.M., Shaper A.G. (1995). Prospective study of serum total homocysteine concentration and risk of stroke in middle-aged British men. Lancet.

[19] Faurschou M., Nielsen O.J., Jensen M.K., Hasselbalch H.C. (2000). High prevalence of hyperhomocysteinemia due to marginal deficiency of cobalamin or folate in chronic myeloproliferative disorders. Am. J. Hematol..

[20] Hultberg B., Agardh C.D., Agardh E., Lovestam-Adrian M. (1997). Poor metabolic control, early age at onset, and marginal folate deficiency are associated with increasing levels of plasma homocysteine in insulin-dependent diabetes mellitus. A five-year follow-up study. Scand. J. Clin. Lab. Investig..

[21] Hultberg B., Andersson A., Lindgren A. (1997). Marginal folate deficiency as a possible cause of hyperhomocystinaemia in stroke patients. Eur. J. Clin. Chem. Clin. Biochem..

[22] Cheng S.W., Ting A.C., Wong J. (1997). Fasting total plasma homocysteine and atherosclerotic peripheral vascular disease. Ann. Vasc. Surg..

[23] Beaumont V., Malinow M.R., Sexton G., Wilson D., Lemort N., Upson B., Beaumont J.L. (1992). Hyperhomocyst(e)inemia, anti-estrogen antibodies and other risk factors for thrombosis in women on oral contraceptives. Atherosclerosis.

[24] Urnov F.D. (2002). Methylation and the genome: The power of a small amendment. J. Nutr..

[25] Zhu B.T. (2002). On the mechanism of homocysteine pathophysiology and pathogenesis: A unifying hypothesis. Histol. Histopathol..

[26] Bautista L.E., Arenas I.A., Peñuela A., Martínez L.X. (2002). Total plasma homocysteine level and risk of cardiovascular disease: A meta-analysis of prospective cohort studies. J. Clin. Epidemiol..

[27] Khare A., Lopez M., Gogtay J. (2006). Homocysteine, B vitamins, and cardiovascular disease. N. Engl. J. Med..

[28] Celik N., Vurmaz A., Kahraman A. (2017). Protective effect of quercetin on homocysteine-induced oxidative stress. Nutrition.

[29] Selhub J. (1999). Homocysteine metabolism. Annu. Rev. Nutr..

[30] Rizzo A., Sciorsci R.L. (2019). Role of homocysteine metabolism in animal reproduction: A review. Res. Vet. Sci..

[31] Rehman T., Shabbir M.A., Inam-Ur-Raheem M., Manzoor M.F., Ahmad N., Liu Z.W., Ahmad M.H., Siddeeg A., Abid M., Aadil R.M. (2020). Cysteine and homocysteine as biomarker of various diseases. Food Sci. Nutr..

[32] Stead L.M., Brosnan J.T., Brosnan M.E., Vance D.E., Jacobs R.L. (2006). Is it time to reevaluate methyl balance in humans?. Am. J. Clin. Nutr..

[33] Carmel R., Gott P.S., Waters C.H., Cairo K., Green R., Bondareff W., DeGiorgio C.M., Cummings J.L., Jacobsen D.W., Buckwalter G. (1995). The frequently low cobalamin levels in dementia usually signify treatable metabolic, neurologic and electrophysiologic abnormalities. Eur. J. Haematol..

[34] Allen R.H., Stabler S.P., Savage D.G., Lindenbaum J. (1993). Metabolic abnormalities in cobalamin (vitamin B12) and folate deficiency. FASEB J..

[35] Galbiatti A.L., Ruiz M.T., Rezende Pinto D., Raposo L.S., Maniglia J.V., Pavarino-Bertelli E.C., Goloni-Bertollo E.M. (2011). A80G polymorphism of reduced folate carrier 1 (RFC1) gene and head and neck squamous cell carcinoma etiology in Brazilian population. Mol. Biol. Rep..

[36] Biselli J.M., Brumati D., Frigeri V.F., Zampieri B.L., Goloni-Bertollo E.M., Pavarino-Bertelli E.C. (2008). A80G polymorphism of reduced folate carrier 1 (RFC1) and C776G polymorphism of transcobalamin 2 (TC2) genes in Down’s syndrome etiology. Sao Paulo Med. J..

[37] Desouza C., Keebler M., McNamara D.B., Fonseca V. (2002). Drugs affecting homocysteine metabolism: Impact on cardiovascular risk. Drugs.

[38] Deedwania P.C. (2013). New oral anticoagulants in elderly patients with atrial fibrillation. Am. J. Med..

[39] Miller D.J., Simpson J.R., Silver B. (2011). Safety of thrombolysis in acute ischemic stroke: A review of complications, risk factors, and newer technologies. Neurohospitalist.

[40] Sacco R.L. (2001). Newer risk factors for stroke. Neurology.

[41] Cotter A.M., Molloy A.M., Scott J.M., Daly S.F. (2003). Elevated plasma homocysteine in early pregnancy: A risk factor for the development of nonsevere preeclampsia. Am. J. Obstet. Gynecol..

[42] Ramlau-Hansen C.H., Moller U.K., Moller J., Thulstrup A.M. (2003). Lactation—A risk factor for elevated plasma homocysteine?. Ugeskr. Laeger.

[43] Hultberg B. (2003). Modulation of extracellular homocysteine concentration in human cell lines. Clin. Chim. Acta.

[44] Sakamoto A., Nishimura Y., Ono H., Sakura N. (2002). Betaine and homocysteine concentrations in foods. Pediatr. Int..

[45] Wang N., Chen M., Gao J., Ji X., He J., Zhang J., Zhao W. (2019). A series of BODIPY-based probes for the detection of cysteine and homocysteine in living cells. Talanta.

[46] Kostic S., Micovic Z., Andrejevic L., Cvetkovic S., Stamenkovic A., Stankovic S., Obrenovic R., Labudovic-Borovic M., Hrncic D., Jakovljevic V. (2019). The effects of L-cysteine and N-acetyl-L-cysteine on homocysteine metabolism and haemostatic markers, and on cardiac and aortic histology in subchronically methionine-treated Wistar male rats. Mol. Cell. Biochem..

[47] Hannibal L., Blom H.J. (2017). Homocysteine and disease: Causal associations or epiphenomenons?. Mol. Asp. Med..

[48] Kolling J., Scherer E.B., da Cunha A.A., da Cunha M.J., Wyse A.T. (2011). Homocysteine induces oxidative-nitrative stress in heart of rats: Prevention by folic acid. Cardiovasc. Toxicol..

[49] Scherer E.B., da Cunha A.A., Kolling J., da Cunha M.J., Schmitz F., Sitta A., Lima D.D., Delwing D., Vargas C.R., Wyse A.T. (2011). Development of an animal model for chronic mild hyperhomocysteinemia and its response to oxidative damage. Int. J. Dev. Neurosci..

[50] Kaplan P., Tatarkova Z., Sivonova M.K., Racay P., Lehotsky J. (2020). Homocysteine and Mitochondria in Cardiovascular and Cerebrovascular Systems. Int. J. Mol. Sci..

[51] Perna A.F., Ingrosso D., Lombardi C., Acanfora F., Satta E., Cesare C.M., Violetti E., Romano M.M., De Santo N.G. (2003). Possible mechanisms of homocysteine toxicity. Kidney Int. Suppl..

[52] Esse R., Barroso M., Tavares de Almeida I., Castro R. (2019). The Contribution of Homocysteine Metabolism Disruption to Endothelial Dysfunction: State-of-the-Art. Int. J. Mol. Sci..

[53] Ostrakhovitch E.A., Tabibzadeh S. (2019). Homocysteine and age-associated disorders. Ageing Res. Rev..

[54] Rossi A., Pizzo P., Filadi R. (2019). Calcium, mitochondria and cell metabolism: A functional triangle in bioenergetics. Biochim. Biophys. Acta Mol. Cell Res..

[55] Lash L.H., Anders M.W. (1987). Mechanism of S-(1,2-dichlorovinyl)-L-cysteine- and S-(1,2-dichlorovinyl)-L-homocysteine-induced renal mitochondrial toxicity. Mol. Pharmacol..

[56] Dos Santos T.M., Siebert C., de Oliveira M.F., Manfredini V., Wyse A.T.S. (2019). Chronic mild Hyperhomocysteinemia impairs energy metabolism, promotes DNA damage and induces a Nrf2 response to oxidative stress in rats brain. Cell. Mol. Neurobiol..

[57] Wyse A.T.S., Sanches E.F., Dos Santos T.M., Siebert C., Kolling J., Netto C.A. (2020). Chronic mild hyperhomocysteinemia induces anxiety-like symptoms, aversive memory deficits and hippocampus atrophy in adult rats: New insights into physiopathological mechanisms. Brain Res..

[58] Kumar M., Sandhir R. (2020). Hydrogen sulfide attenuates hyperhomocysteinemia-induced mitochondrial dysfunctions in brain. Mitochondrion.

[59] Folbergrova J., Jesina P., Drahota Z., Lisy V., Haugvicova R., Vojtiskova A., Houstek J. (2007). Mitochondrial complex I inhibition in cerebral cortex of immature rats following homocysteic acid-induced seizures. Exp. Neurol..

[60] Bhattacharjee N., Borah A. (2016). Oxidative stress and mitochondrial dysfunction are the underlying events of dopaminergic neurodegeneration in homocysteine rat model of Parkinson’s disease. Neurochem. Int..

[61] Kolling J., Scherer E.B., Siebert C., Longoni A., Loureiro S., Weis S., Petenuzzo L., Wyse A.T. (2016). Severe Hyperhomocysteinemia Decreases Respiratory Enzyme and Na(+)-K(+) ATPase Activities, and Leads to Mitochondrial Alterations in Rat Amygdala. Neurotox. Res..

[62] Folbergrova J., Jesina P., Haugvicova R., Lisy V., Houstek J. (2010). Sustained deficiency of mitochondrial complex I activity during long periods of survival after seizures induced in immature rats by homocysteic acid. Neurochem. Int..

[63] Bhattacharjee N., Paul R., Giri A., Borah A. (2016). Chronic exposure of homocysteine in mice contributes to dopamine loss by enhancing oxidative stress in nigrostriatum and produces behavioral phenotypes of Parkinson’s disease. Biochem. Biophys. Rep..

[64] Mishra D.K., Gautam S., Goyal B.K., Kawar R. (2013). Hyperhomocysteinemia as a cause of left main artery thrombosis manifesting as extensive anterior wall MI in a 10 year old girl. J. Assoc. Physicians India.

[65] Mishra P.K., Tyagi N., Sen U., Joshua I.G., Tyagi S.C. (2010). Synergism in hyperhomocysteinemia and diabetes: Role of PPAR gamma and tempol. Cardiovasc. Diabetol..

[66] Finkelstein J.D. (1990). Methionine metabolism in mammals. J. Nutr. Biochem..

[67] Frosst P., Blom H.J., Milos R., Goyette P., Sheppard C.A., Matthews R.G., Boers G.J., den Heijer M., Kluijtmans L.A., van den Heuvel L.P. (1995). A candidate genetic risk factor for vascular disease: A common mutation in methylenetetrahydrofolate reductase. Nat. Genet..

[68] Bailey L.B., Gregory J.F. (1999). Polymorphisms of methylenetetrahydrofolate reductase and other enzymes: Metabolic significance, risks and impact on folate requirement. J. Nutr..

[69] Refsum H., Ueland P.M., Nygard O., Vollset S.E. (1998). Homocysteine and cardiovascular disease. Annu. Rev. Med..

[70] Warren C.J. (2002). Emergent cardiovascular risk factor: Homocysteine. Prog. Cardiovasc. Nurs..

[71] Medina M.A., Amores-Sanchez M.I. (2000). Homocysteine: An emergent cardiovascular risk factor?. Eur. J. Clin. Invest..

[72] Jiang S., Pan M., Wu S., Venners S.A., Zhong G., Hsu Y.H., Weinstock J., Wang B., Tang G., Liu D. (2016). Elevation in Total Homocysteine Levels in Chinese Patients with Essential Hypertension Treated with Antihypertensive Benazepril. Clin. Appl. Thromb. Hemost..

[73] Muller T., Jugel C., Ehret R., Ebersbach G., Bengel G., Muhlack S., Klostermann F. (2011). Elevation of total homocysteine levels in patients with Parkinson’s disease treated with duodenal levodopa/carbidopa gel. J. Neural Transm. (Vienna).

[74] Stabler S.P., Marcell P.D., Podell E.R., Allen R.H., Savage D.G., Lindenbaum J. (1988). Elevation of total homocysteine in the serum of patients with cobalamin or folate deficiency detected by capillary gas chromatography-mass spectrometry. J. Clin. Invest..

[75] Selhub J., Miller J.W. (1992). The pathogenesis of homocysteinemia: Interruption of the coordinate regulation by S-adenosylmethionine of the remethylation and transsulfuration of homocysteine. Am. J. Clin. Nutr..

[76] Cheng D., Kong H., Pang W., Yang H., Lu H., Huang C., Jiang Y. (2016). B vitamin supplementation improves cognitive function in the middle aged and elderly with hyperhomocysteinemia. Nutr. Neurosci..

[77] Clarke R. (2011). Homocysteine, B vitamins, and the risk of cardiovascular disease. Clin. Chem..

[78] Blacher J., Czernichow S., Horrellou M.H., Conad J., David P., Chadefaux-Vekemans B., Ankria A., Galan P., Hercberg S., Ducimetiere P. (2005). Homocysteine, folic acid, group B vitamins and cardiovascular risk. Arch. Mal. Coeur Vaiss..

[79] Zhuo J.M., Wang H., Pratico D. (2011). Is hyperhomocysteinemia an Alzheimer’s disease (AD) risk factor, an AD marker, or neither?. Trends Pharmacol. Sci..

[80] Carmel R., Green R., Rosenblatt D.S., Watkins D. (2003). Update on cobalamin, folate, and homocysteine. Hematology.

[81] Ansari R., Mahta A., Mallack E., Luo J.J. (2014). Hyperhomocysteinemia and neurologic disorders: A review. J. Clin. Neurol..

[82] Choe Y.M., Sohn B.K., Choi H.J., Byun M.S., Seo E.H., Han J.Y., Kim Y.K., Yoon E.J., Lee J.M., Park J. (2014). Association of homocysteine with hippocampal volume independent of cerebral amyloid and vascular burden. Neurobiol. Aging.

[83] Skovierova H., Vidomanova E., Mahmood S., Sopkova J., Drgova A., Cervenova T., Halasova E., Lehotsky J. (2016). The Molecular and Cellular Effect of Homocysteine Metabolism Imbalance on Human Health. Int. J. Mol. Sci..

[84] Kamat P.K., Vacek J.C., Kalani A., Tyagi N. (2015). Homocysteine Induced Cerebrovascular Dysfunction: A Link to Alzheimer’s Disease Etiology. Open Neurol. J..

[85] Tyagi N., Gillespie W., Vacek J.C., Sen U., Tyagi S.C., Lominadze D. (2009). Activation of GABA-A receptor ameliorates homocysteine-induced MMP-9 activation by ERK pathway. J. Cell. Physiol..

[86] Zhang C.E., Tian Q., Wei W., Peng J.H., Liu G.P., Zhou X.W., Wang Q., Wang D.W., Wang J.Z. (2008). Homocysteine induces tau phosphorylation by inactivating protein phosphatase 2A in rat hippocampus. Neurobiol. Aging.

[87] Chen S., Dong Z., Cheng M., Zhao Y., Wang M., Sai N., Wang X., Liu H., Huang G., Zhang X. (2017). Homocysteine exaggerates microglia activation and neuroinflammation through microglia localized STAT3 overactivation following ischemic stroke. J. Neuroinflamm..

[88] Fan X., Zhang L., Li H., Chen G., Qi G., Ma X., Jin Y. (2020). Role of homocysteine in the development and progression of Parkinson’s disease. Ann. Clin. Transl. Neurol..

[89] Zieminska E., Lazarewicz J.W. (2006). Excitotoxic neuronal injury in chronic homocysteine neurotoxicity studied in vitro: The role of NMDA and group I metabotropic glutamate receptors. Acta Neurobiol. Exp. (Wars).

[90] Valkovic P., Benetin J., Blazicek P., Valkovicova L., Gmitterova K., Kukumberg P. (2005). Reduced plasma homocysteine levels in levodopa/entacapone treated Parkinson patients. Parkinsonism Relat. Disord..

[91] Racek J., Rusnakova H., Trefil L., Siala K.K. (2005). The influence of folate and antioxidants on homocysteine levels and oxidative stress in patients with hyperlipidemia and hyperhomocysteinemia. Physiol. Res..

[92] Lee S.H., Kim M.J., Kim B.J., Kim S.R., Chun S., Ryu J.S., Kim G.S., Lee M.C., Koh J.M., Chung S.J. (2010). Homocysteine-lowering therapy or antioxidant therapy for bone loss in Parkinson’s disease. Mov. Disord..

[93] Johnson N.L., Burkett K., Reinhold J., Bultas M.W. (2016). Translating Research to Practice for Children with Autism Spectrum Disorder: Part I: Definition, Associated Behaviors, Prevalence, Diagnostic Process, and Interventions. J. Pediatr. Health Care.

[94] Yoo H. (2015). Genetics of Autism Spectrum Disorder: Current Status and Possible Clinical Applications. Exp. Neurobiol..

[95] Subramanian M., Timmerman C.K., Schwartz J.L., Pham D.L., Meffert M.K. (2015). Characterizing autism spectrum disorders by key biochemical pathways. Front. Neurosci..

[96] Kiykim E., Zeybek C.A., Zubarioglu T., Cansever S., Yalcinkaya C., Soyucen E., Aydin A. (2016). Inherited metabolic disorders in Turkish patients with autism spectrum disorders. Autism Res..

[97] James S.J., Cutler P., Melnyk S., Jernigan S., Janak L., Gaylor D.W., Neubrander J.A. (2004). Metabolic biomarkers of increased oxidative stress and impaired methylation capacity in children with autism. Am. J. Clin. Nutr..

[98] Tu W.J., Yin C.H., Guo Y.Q., Li S.O., Chen H., Zhang Y., Feng Y.L., Long B.H. (2013). Serum homocysteine concentrations in Chinese children with autism. Clin. Chem. Lab. Med..

[99] Ali A., Waly M.I., Al-Farsi Y.M., Essa M.M., Al-Sharbati M.M., Deth R.C. (2011). Hyperhomocysteinemia among Omani autistic children: A case-control study. Acta Biochim. Pol..

[100] Kaluzna-Czaplinska J., Michalska M., Rynkowski J. (2011). Homocysteine level in urine of autistic and healthy children. Acta Biochim. Pol..

[101] Ghanizadeh A. (2013). Increased glutamate and homocysteine and decreased glutamine levels in autism: A review and strategies for future studies of amino acids in autism. Dis. Markers.

[102] Kral T.V., Eriksen W.T., Souders M.C., Pinto-Martin J.A. (2013). Eating behaviors, diet quality, and gastrointestinal symptoms in children with autism spectrum disorders: A brief review. J. Pediatr. Nurs..

[103] Ranjan S., Nasser J.A. (2015). Nutritional status of individuals with autism spectrum disorders: Do we know enough?. Adv. Nutr..

[104] McGrath J., Saha S., Welham J., El Saadi O., MacCauley C., Chant D. (2004). A systematic review of the incidence of schizophrenia: The distribution of rates and the influence of sex, urbanicity, migrant status and methodology. BMC Med..

[105] Arroll M.A., Wilder L., Neil J. (2014). Nutritional interventions for the adjunctive treatment of schizophrenia: A brief review. Nutr. J..

[106] Vita A., Barlati S., De Peri L., Deste G., Sacchetti E. (2016). Schizophrenia. Lancet.

[107] Regland B., Johansson B.V., Grenfeldt B., Hjelmgren L.T., Medhus M. (1995). Homocysteinemia is a common feature of schizophrenia. J. Neural Transm. Gen. Sect..

[108] Regland B., Johansson B.V., Gottfries C.G. (1994). Homocysteinemia and schizophrenia as a case of methylation deficiency. J. Neural Transm. Gen. Sect..

[109] Muntjewerff J.W., Kahn R.S., Blom H.J., den Heijer M. (2006). Homocysteine, methylenetetrahydrofolate reductase and risk of schizophrenia: A meta-analysis. Mol. Psychiatry.

[110] Kim T.H., Moon S.W. (2011). Serum homocysteine and folate levels in korean schizophrenic patients. Psychiatry Investig..

[111] Di Lorenzo R., Amoretti A., Baldini S., Soli M., Landi G., Pollutri G., Corradini R., Ferri P. (2015). Homocysteine levels in schizophrenia patients newly admitted to an acute psychiatric ward. Acta Neuropsychiatr..

[112] Ayesa-Arriola R., Perez-Iglesias R., Rodriguez-Sanchez J.M., Mata I., Gomez-Ruiz E., Garcia-Unzueta M., Martinez-Garcia O., Tabares-Seisdedos R., Vazquez-Barquero J.L., Crespo-Facorro B. (2012). Homocysteine and cognition in first-episode psychosis patients. Eur. Arch. Psychiatry Clin. Neurosci..

[113] Kinoshita M., Numata S., Tajima A., Nishi A., Muraki S., Tsuchiya A., Umehara H., Watanabe S.Y., Imoto I., Ohmori T. (2016). Cumulative effect of the plasma total homocysteine-related genetic variants on schizophrenia risk. Psychiatry Res..

[114] Numata S., Kinoshita M., Tajima A., Nishi A., Imoto I., Ohmori T. (2015). Evaluation of an association between plasma total homocysteine and schizophrenia by a Mendelian randomization analysis. BMC Med. Genet..

[115] Kinoshita M., Numata S., Tajima A., Shimodera S., Imoto I., Ohmori T. (2013). Plasma total homocysteine is associated with DNA methylation in patients with schizophrenia. Epigenetics.

[116] Chia S.C., Henry J., Mok Y.M., Honer W.G., Sim K. (2015). Fatty acid and vitamin interventions in adults with schizophrenia: A systematic review of the current evidence. J. Neural Transm. (Vienna).

[117] Roffman J.L., Lamberti J.S., Achtyes E., Macklin E.A., Galendez G.C., Raeke L.H., Silverstein N.J., Smoller J.W., Hill M., Goff D.C. (2013). Randomized multicenter investigation of folate plus vitamin B12 supplementation in schizophrenia. JAMA Psychiatry.

[118] Lacerda A.L., Keshavan M.S., Hardan A.Y., Yorbik O., Brambilla P., Sassi R.B., Nicoletti M., Mallinger A.G., Frank E., Kupfer D.J. (2004). Anatomic evaluation of the orbitofrontal cortex in major depressive disorder. Biol. Psychiatry.

[119] Hasler G. (2010). Pathophysiology of depression: Do we have any solid evidence of interest to clinicians?. World Psychiatry.

[120] Copeland W.E., Adair C.E., Smetanin P., Stiff D., Briante C., Colman I., Fergusson D., Horwood J., Poulton R., Costello E.J. (2013). Diagnostic transitions from childhood to adolescence to early adulthood. J. Child Psychol. Psychiatry.

[121] Djernes J.K. (2006). Prevalence and predictors of depression in populations of elderly: A review. Acta Psychiatr. Scand..

[122] Bottiglieri T., Laundy M., Crellin R., Toone B.K., Carney M.W., Reynolds E.H. (2000). Homocysteine, folate, methylation, and monoamine metabolism in depression. J. Neurol. Neurosurg. Psychiatry.

[123] Cosar A., Ipcioglu O.M., Ozcan O., Gultepe M. (2014). Folate and homocysteine metabolisms and their roles in the biochemical basis of neuropsychiatry. Turk. J. Med. Sci..

[124] Baek J.H., Bernstein E.E., Nierenberg A.A. (2013). One-carbon metabolism and bipolar disorder. Aust. N. Z. J. Psychiatry.

[125] Coppen A., Bolander-Gouaille C. (2005). Treatment of depression: Time to consider folic acid and vitamin B12. J. Psychopharmacol..

[126] Lee E.S.Y., Chen H.T., Soliman K.F.A., Charlton C.G. (2005). Effects of homocysteine on the dopaminergic system and behavior in rodents. Neurotoxicology.

[127] Berk M., Dodd S., Kauer-Sant’Anna M., Malhi G.S., Bourin M., Kapczinski F., Norman T. (2007). Dopamine dysregulation syndrome: Implications for a dopamine hypothesis of bipolar disorder. Acta Psychiatr. Scand..

[128] Ghanizadeh A., Singh A.B., Berk M., Torabi-Nami M. (2015). Homocysteine as a potential biomarker in bipolar disorders: A critical review and suggestions for improved studies. Expert Opin. Ther. Targets.

[129] Permoda-Osip A., Dorszewska J., Skibinska M., Chlopocka-Wozniak M., Rybakowski J.K. (2013). Hyperhomocysteinemia in Bipolar Depression: Clinical and Biochemical Correlates. Neuropsychobiology.

[130] Moustafa A.A., Hewedi D.H., Eissa A.M., Frydecka D., Misiak B. (2014). Homocysteine levels in schizophrenia and affective disorders—focus on cognition. Front. Behav. Neurosci..

[131] Ezzaher A., Mouhamed D.H., Mechri A., Omezzine A., Neffati F., Douki W., Bouslama A., Gaha L., Najjar M.F. (2011). Hyperhomocysteinemia in Tunisian bipolar I patients. Psychiatry Clin. Neurosci..

[132] Rodrigo C., De Silva N.L., Gunaratne R., Rajapakse S., De Silva V.A., Hanwella R. (2015). Lower Estimated Glomerular Filtration Rates in Patients on Long Term Lithium; a Comparative Study and a Meta-analysis of Literature. Eur. Psychiatry.

[133] Enderle J., Klink U., di Giuseppe R., Koch M., Seidel U., Weber K., Birringer M., Ratjen I., Rimbach G., Lieb W. (2020). Plasma Lithium Levels in the General Population: A Cross-Sectional Analysis of Metabolic and Dietary Correlates. Nutrients.

[134] Ubeda N., Alonso-Aperte E., Varela-Moreiras G. (2002). Acute valproate administration impairs methionine metabolism in rats. J. Nutr..

[135] Lowenstein C.J., Dinerman J.L., Snyder S.H. (1994). Nitric oxide: A physiologic messenger. Ann. Intern. Med..

[136] Narayan S.K., Verman A., Kattimani S., Ananthanarayanan P.H., Adithan C. (2014). Plasma homocysteine levels in depression and schizophrenia in South Indian Tamilian population. Indian J. Psychiatry.

[137] Misiak B., Frydecka D., Slezak R., Piotrowski P., Kiejna A. (2014). Elevated homocysteine level in first-episode schizophrenia patients—The relevance of family history of schizophrenia and lifetime diagnosis of cannabis abuse. Metab. Brain Dis..

[138] Ma Y.Y., Shek C.C., Wong M.C., Yip K.C., Ng R.M., Nguyen D.G., Poon T.K. (2009). Homocysteine level in schizophrenia patients. Aust. N. Z. J. Psychiatry.

[139] Nishi A., Numata S., Tajima A., Kinoshita M., Kikuchi K., Shimodera S., Tomotake M., Ohi K., Hashimoto R., Imoto I. (2014). Meta-analyses of blood homocysteine levels for gender and genetic association studies of the MTHFR C677T polymorphism in schizophrenia. Schizophr. Bull..

[140] Kevere L., Purvina S., Bauze D., Zeibarts M., Andrezina R., Rizevs A., Jelisejevs S., Piekuse L., Kreile M., Purvins I. (2012). Elevated serum levels of homocysteine as an early prognostic factor of psychiatric disorders in children and adolescents. Schizophr. Res. Treat..

[141] Li J.B., Cheng Y.C., Shi M., Tang J.R., Dai Q., Zhang Y., Chen J.W., Wang H.X. (2011). Association of homocysteine with peripheral neuropathy in Chinese patients with type 2 diabetes. Diabetes Res. Clin. Pract..

[142] Belardo A., Gevi F., Zolla L. (2019). The concomitant lower concentrations of vitamins B6, B9 and B12 may cause methylation deficiency in autistic children. J. Nutr. Biochem..

[143] Weir D.G., Keating S., Molloy A., Mcpartlin J., Kennedy S., Blanchflower J., Kennedy D.G., Rice D., Scott J.M. (1988). Methylation Deficiency Causes Vitamin-B12-Associated Neuropathy in the Pig. J. Neurochem..

[144] Varshney K.K., Gupta J.K., Mujwar S. (2019). Homocysteine Induced Neurological Dysfunctions: A Link to Neurodegenerative Disorders. Int. J. Med. Res. Health Sci..

[145] Faverzani J.L., Hammerschmidt T.G., Sitta A., Deon M., Wajner M., Vargas C.R. (2017). Oxidative Stress in Homocystinuria Due to Cystathionine -Synthase Deficiency: Findings in Patients and in Animal Models. Cell. Mol. Neurobiol..

[146] Holmes M.V., Newcombe P., Hubacek J.A., Sofat R., Ricketts S.L., Cooper J., Breteler M.M.B., Bautista L.E., Sharma P., Whittaker J.C. (2011). Effect modification by population dietary folate on the association between MTHFR genotype, homocysteine, and stroke risk: A meta-analysis of genetic studies and randomised trials. Lancet.

[147] Casas J.P., Bautista L.E., Smeeth L., Sharma P., Hingorani A.D. (2005). Homocysteine and stroke: Evidence on a causal link from mendelian randomisation. Lancet.

[148] Casas J.P., Bautista L.E., Hingorani A.D., Sharma P. (2004). Plasma homocysteine, and ischaemic stroke: “Mendelian randomization” provides further evidence of causal link. J. Neurol. Neurosurg. Psychiatry.

[149] Lehotsky J., Tothova B., Kovalska M., Dobrota D., Benova A., Kalenska D., Kaplan P. (2016). Role of Homocysteine in the Ischemic Stroke and Development of Ischemic Tolerance. Front. Neurosci..

[150] Poddar R., Paul S. (2009). Homocysteine-NMDA receptor-mediated activation of extracellular signal-regulated kinase leads to neuronal cell death. J. Neurochem..

[151] Gorgone G., Caccamo D., Pisani L.R., Curro M., Parisi G., Oteri G., Ientile R., Rossini P.M., Pisani F. (2009). Hyperhomocysteinemia in patients with epilepsy: Does it play a role in the pathogenesis of brain atrophy? A preliminary report. Epilepsia.

[152] Caccamo D., Condello S., Gorgone G., Crisafulli G., Belcastro V., Gennaro S., Striano P., Pisani F., Ientile R. (2004). Screening for C677T and A1298C MTHFR polymorphisms in patients with epilepsy and risk of hyperhomocysteinemia. Neuromol. Med..

[153] Folbergrova J. (1997). Anticonvulsant action of both NMDA and Non-NMDA receptor antagonists against seizures induced by homocysteine in immature rats. Exp. Neurol..

[154] Diaz-Arrastia R. (2000). Homocysteine and neurologic disease. Arch. Neurol..

[155] Hamed S.A. (2009). The vascular risk associations with migraine: Relation to migraine susceptibility and progression. Atherosclerosis.

[156] Kara I., Sazci A., Ergul E., Kaya G., Kilic G. (2003). Association of the C677T and A1298C polymorphisms in the 5,10 methylenetetrahydrofolate reductase gene in patients with migraine risk. Mol. Brain Res..

[157] Cacciapuoti F. (2017). Migraine homocysteine-related: Old and new mechanisms. Neurol. Clin. Neurosci..

[158] Al-Qasmi M.M., Athanas K., Dafer R.M., Tietjen G.E. (2000). Von Willebrand Factor is elevated in migraineurs with aura, transient ischemic attacks, and stroke: A retrospective analysis. Neurology.

[159] Hering-Hanit R., Friedman Z., Schlesinger I., Ellis M. (2001). Evidence for activation of the coagulation system in migraine with aura. Cephalalgia.

[160] Scher A.I., Terwindt G.M., Verschuren W.M.M., Kruit M.C., Blom H.J., Kowa H., Frants R.R., Maagdenberg A.M.J.M.V.D., A Van Buchem M., Ferrari M.D. (2006). Migraine and MTHFR C677T genotype in a population-based sample. Ann. Neurol. Off. J. Am. Neurol. Assoc. Child Neurol. Soc..

[161] Hering-Hanit R., Friedman Z., Schlesinger I., Ellis M. (1999). Activation of the coagulation system in migraine with aura. Cephalalgia.

[162] Edvinsson L., Uddman R. (2005). Neurobiology in primary headaches. Brain Res. Rev..

[163] Liampas I., Siokas V., Mentis A.A., Aloizou A., Dastamani M., Tsouris Z., Aslanidou P., Brotis A., Dardiotis E. (2020). Serum Homocysteine, Pyridoxine, Folate, and Vitamin B12 Levels in Migraine: Systematic Review and Meta-Analysis. Headache J. Head Face Pain.

[164] Goadsby P.J. (2015). Migraine and other primary headache disorders. Pain: The Person, the Science, the Clinical Interface.

[165] Smith A.D., Kim Y.I., Refsum H. (2008). Is folic acid good for everyone?. Am. J. Clin. Nutr..

[166] Goodwin G.M., Consensus Group of the British Association for Psychopharmacology (2009). Evidence-based guidelines for treating bipolar disorder: Revised second edition—recommendations from the British Association for Psychopharmacology. J. Psychopharmacol..

[167] Merikangas K.R., Akiskal H.S., Angst J., Greenberg P.E., Hirschfeld R.M.A., Petukhova M., Kessler R.C. (2007). Lifetime and 12-month prevalence of bipolar spectrum disorder in the national comorbidity survey replication. Arch. Gen. Psychiatry.

[168] Merikangas K.R., Jin R., He J.P., Kessler R.C., Lee S., Sampson N.A., Viana M.C., Andrade L.H., Hu C.Y., Karam E.G. (2011). Prevalence and Correlates of Bipolar Spectrum Disorder in the World Mental Health Survey Initiative. Arch. Gen. Psychiatry.

[169] Blaise S.A., Nedelec E., Alberto J.M., Schroeder H., Audonnet S., Bossenmeyer-Pourie C., Gueant J.L., Daval J.L. (2009). Short hypoxia could attenuate the adverse effects of hyperhomocysteinemia on the developing rat brain by inducing neurogenesis. Exp. Neurol..

[170] Hrncic D., Rasic-Markovic A., Lekovic J., Krstic D., Colovic M., Macut D., Susic V., Djuric D., Stanojlovic O. (2014). Exercise Decreases Susceptibility to Homocysteine Seizures: The Role of Oxidative Stress. Int. J. Sports Med..

[171] Oterino A., Valle N., Bravo Y., Munoz P., Sanchez-Velasco P., Ruiz-Alegria C., Castillo J., Leyva-Cobian F., Vadillo A., Pascual J. (2004). MTHFR T677 homozygosis influences the presence of aura in migraineurs. Cephalalgia.

[172] Cacciapuoti F. (2012). Lowering homocysteine levels may prevent cardiovascular impairments? Possible therapeutic behaviors. Blood Coagul. Fibrin..

[173] Lippi G., Mattiuzzi C., Meschi T., Cervellin G., Borghi L. (2014). Homocysteine and migraine. A narrative review. Clin. Chim. Acta.

[174] Refsum H., Smith A.D., Ueland P.M., Nexo E., Clarke R., McPartlin J., Johnston C., Engbaek F., Schneede J., McPartlin C. (2004). Facts and recommendations about total homocysteine determinations: An expert opinion. Clin. Chem..

[175] Kim S., Lim I.K., Park G.H., Paik W.K. (1997). Biological methylation of myelin basic protein: Enzymology and biological significance. Int. J. Biochem. Cell B.

[176] Reynolds E. (2006). Vitamin B12, folic acid, and the nervous system. Lancet Neurol..

[177] Kruman I.I., Culmsee C., Chan S.L., Kruman Y., Guo Z.H., Penix L., Mattson M.P. (2000). Homocysteine elicits a DNA damage response in neurons that promotes apoptosis and hypersensitivity to excitotoxicity. J. Neurosci..

[178] Sengupta S., Wehbe C., Majors A.K., Ketterer M.E., DiBello P.M., Jacobsen D.W. (2001). Relative roles of albumin and ceruloplasmin in the formation of homocystine, homocysteine-cysteine-mixed disulfide, and cystine in circulation. J. Biol. Chem..

[179] Au-Yeung K.K.W., Yip J.C.W., Siow Y.L., Karmin O. (2006). Folic acid inhibits homocysteine-induced superoxide anion production and nuclear factor kappa B activation in macrophages. Can. J. Physiol. Pharm..

[180] Tajouri L., Martin V., Gasparini C., Ovcaric M., Curtain R., Lea R.A., Haupt L.M., Csurhes P., Pender M.P., Griffiths L.R. (2006). Genetic investigation of methylenetetrahydrofolate reductase (MTHFR) and catechol-O-methyl transferase (COMT) in multiple sclerosis. Brain Res. Bull..

[181] Kang S.S., Zhou J., Wong P.W.K., Kowalisyn J., Strokosch G. (1988). Intermediate Homocysteinemia—A Thermolabile Variant of Methylenetetrahydrofolate Reductase. Am. J. Hum. Genet..

[182] Szvetko A.L., Fowdar J., Nelson J., Colson N., Tajouri L., Csurhes P.A., Pender M.P., Griffiths L.R. (2007). No association between MTHFR A1298C and MTRR A66G polymorphisms, and MS in an Australian cohort. J. Neurol. Sci..

[183] Oliveira S.R., Flauzino T., Sabino B.S., Kallaur A.P., Alfieri D.F., Kaimen-Maciel D.R., Morimoto H.K., de Almeida E.R.D., Lozovoy M.A.B., Reiche E.M.V. (2018). Elevated plasma homocysteine levels are associated with disability progression in patients with multiple sclerosis. Metab. Brain Dis..

[184] Kararizou E., Paraskevas G., Triantafyllou N., Koutsis G., Evangelopoulos M.E., Mandellos D., Sfagos C., Kapaki E. (2013). Plasma homocysteine levels in patients with multiple sclerosis in the Greek population. J. Chin. Med Assoc..

[185] Zoccolella S., Tortorella C., Iaffaldano P., Direnzo V., D’Onghia M., Paolicelli D., Livrea P., Trojano M. (2012). Elevated plasma homocysteine levels in patients with multiple sclerosis are associated with male gender. J. Neurol..

[186] Akanji A.O., Al-Shammri S. (2008). Plasma homocysteine and C-reactive protein levels in relation to disease progression in multiple sclerosis. Clin. Chem..

[187] Sahin S., Aksungar F., Topkaya A., Yildiz Z., Boru U., Ayalpl S., Karsidag S. (2007). Increased plasma homocysteine levels in multiple sclerosis. Mult. Scler. J..

[188] Ramsaransing G.M., Fokkema M.R., Teelken A., Arutiunyan A.V., Koch M., De Keyser J. (2006). Plasma homocysteine levels in multiple sclerosis. J. Neurol. Neurosur. Psychiatry.

[189] Besler H.T., Comoglu S. (2003). Lipoprotein oxidation, plasma total antioxidant capacity and homocysteine level in patients with multiple sclerosis. Nutr. Neurosci..

[190] Vrethem M., Mattsson E., Hebelka H., Leerbeck K., Osterberg A., Landtblom A.M., Balla B., Nilsson H., Hultgren M., Brattstrom L. (2003). Increased plasma homocysteine levels without signs of vitamin B-12 deficiency in patients with multiple sclerosis assessed by blood and cerebrospinal fluid homocysteine and methylmalonic acid. Mult. Scler..

[191] Li X.T., Yuan J.L., Han J.M., Hu W.L. (2020). Serum levels of Homocysteine, Vitamin B12 and Folate in Patients with Multiple Sclerosis: An Updated Meta-Analysis. Int. J. Med Sci..

[192] Onar M.K., Yazici T. (2018). Serum Homocysteine Levels and Cognitive Dysfunction in Multiple Sclerosis Patients. Mult. Scler. J..

[193] Fahmy E.M., Elfayoumy N.M., Abdelalim A.M., Sharaf S.A.A., Ismail R.S., Elshebawy H. (2018). Relation of serum levels of homocysteine, vitamin B12 and folate to cognitive functions in multiple sclerosis patients. Int. J. Neurosci..

[194] Kocer B., Engur S., Ak F., Yilmaz M. (2009). Serum vitamin B12, folate, and homocysteine levels and their association with clinical and electrophysiological parameters in multiple sclerosis. J. Clin. Neurosci..

[195] Teunissen C.E., Killestein J., Kragt J.J., Polman C.H., Dijkstra C.D., Blom H.J. (2008). Serum homocysteine levels in relation to clinical progression in multiple sclerosis. J. Neurol. Neurosur. Psychiatry.

[196] Ashtari F., Shaygannejad V. (2006). Serum homocysteine level in patients with multiple sclerosis. Eur. J. Neurol..

[197] Rio J., Montalban J., Tintore M., Codina A., Malinow M.R. (1994). Serum Homocysteine Levels in Multiple-Sclerosis. Arch. Neurol. Chic..

[198] Stabler S.P., Estacio R., Jeffers B.W., Cohen J.A., Allen R.H., Schrier R.W. (1999). Total homocysteine is associated with nephropathy in non-insulin-dependent diabetes mellitus. Metabolism.

[199] Cronin C.C., McPartlin J.M., Barry D.G., Ferriss J.B., Scott J.M., Weir D.G. (1998). Plasma homocysteine concentrations in patients with type 1 diabetes. Diabetes Care.

[200] Ambrosch A., Dierkes J., Lobmann R., Kuhne W., Konig W., Luley C., Lehnert H. (2001). Relation between homocysteinaemia and diabetic neuropathy in patients with Type 2 diabetes mellitus. Diabet. Med..

[201] Buysschaert M., Dramais A.S., Wallemacq P.E., Hermans M.P. (2000). Hyperhomocysteinemia in type 2 diabetes: Relationship to macroangiopathy, nephropathy, and insulin resistance. Diabetes Care.

[202] Chico A., Perez A., Cordoba A., Arcelus R., Carreras G., de Leiva A., Gonzalez-Sastre F., Blanco-Vaca F. (1998). Plasma homocysteine is related to albumin excretion rate in patients with diabetes mellitus: A new link between diabetic nephropathy and cardiovascular disease?. Diabetologia.

[203] Hoogeveen E.K., Kostense P.J., Valk G.D., Bertelsmann F.W., Jakobs C., Dekker J.M., Nijpels G., Heine R.J., Bouter L.M., Stehouwer C.D. (1999). Hyperhomocysteinaemia is not related to risk of distal somatic polyneuropathy: The Hoorn Study. J. Intern. Med..

[204] Ganapathy P.S., White R.E., Ha Y., Bozard B.R., McNeil P.L., Caldwell R.W., Kumar S., Black S.M., Smith S.B. (2011). The role of N-methyl-D-aspartate receptor activation in homocysteine-induced death of retinal ganglion cells. Invest. Ophthalmol. Vis. Sci..

[205] Lipton S.A., Kim W.K., Choi Y.B., Kumar S., D’Emilia D.M., Rayudu P.V., Arnelle D.R., Stamler J.S. (1997). Neurotoxicity associated with dual actions of homocysteine at the N-methyl-D-aspartate receptor. Proc. Natl. Acad. Sci. USA.

[206] Chmiel-Perzynska I., Perzynski A., Wielosz M., Urbanska E.M. (2007). Hyperglycemia enhances the inhibitory effect of mitochondrial toxins and D,L-homocysteine on the brain production of kynurenic acid. Pharmacol. Rep..

[207] Munipally P.K., Agraharm S.G., Valavala V.K., Gundae S., Turlapati N.R. (2011). Evaluation of indoleamine 2,3-dioxygenase expression and kynurenine pathway metabolites levels in serum samples of diabetic retinopathy patients. Arch. Physiol. Biochem..

